# EORTC/ESTRO defined induced oligopersistence of liver metastases from colorectal cancer - outcomes and toxicity profile of computer tomography guided high-dose-rate brachytherapy

**DOI:** 10.1007/s10585-025-10348-z

**Published:** 2025-05-12

**Authors:** Paweł Cisek, Mateusz Bilski, Julia Ponikowska, Ewa Wojtyna, Jacek Fijuth, Łukasz Kuncman

**Affiliations:** 1 Department of Brachytherapy, Saint John’s Cancer Center, Lublin, Poland; 2 Department of Radiotherapy, Medical University of Lublin, Lublin, Poland; 3 Department of Radiotherapy, Saint John’s Cancer Center, Lublin, Poland; 4 Department of Medical Physics, Saint John’s Cancer Center, Lublin, Poland; 5 Department of Radiotherapy, Medical University of Lodz, Łódź, Poland; 6 Department of External Beam Radiotherapy, Copernicus Memorial Hospital in Lodz Comprehensive Cancer Center and Traumatology, Copernicus Memorial Hospital, Pabianicka 62, Łódź, PL 93-513 Poland

**Keywords:** Liver metastases, Brachytherapy, Interventional radiotherapy, Oligometastatic disease, Metastasis directed therapy, Oligopersistent

## Abstract

Colorectal cancer (CRC) often leads to liver metastases, which may be resistant to systemic therapy. This study assessed outcomes and toxicity of computed tomography (CT) guided high-dose-rate (HDR) brachytherapy (BRT) in oligopersistent liver metastases from CRC. The study included patients with liver metastases classified as EORTC/ESTRO-defined induced oligopersistence after multiple systemic therapy lines. Up to four persistent liver metastases per patient were treated with CT-guided brachytherapy (CT-BRT). Treatment response was evaluated using the Response Evaluation Criteria in Solid Tumors (RECIST 1.1). The analysis focused on overall survival (OS), progression-free survival (PFS), tumor burden score (TBS), and the prognostic value of changes in metastasis size. Sixty-eight CRC patients were enrolled. During a median follow-up of 17 months, the median OS was 16 months, and the median PFS was 13 months. Complete response (CR), partial response (PR), stable disease (SD), and progressive disease (PD) were observed in 7%, 35%, 44%, and 6% of patients, respectively. Patients with an objective response (ORR) of 42% had longer OS and PFS than those without it. OS was affected by lymph node metastases and metastasis size reduction, while PFS was additionally influenced by the administered dose. Multivariate analysis showed OS was linked to lymph node metastases (*p* = 0.001) and ORR (*p* = 0.004), and PFS to tumor burden score (TBS) difference (*p* = 0.017) and post-CT-BRT single metastasis size (*p* = 0.026). CT-BRT for CRC oligopersistent liver metastases is effective, improving PFS and OS, with TBS difference identified as a key response parameter for future strategies.

**Supplementary Information:**

The online version contains supplementary material available at 10.1007/s10585-025-10348-z.

## Introduction

Liver metastases from colorectal cancer (CRC) affect 25–30% of patients diagnosed with this condition [1]. Surgical resection is the standard treatment for these patients, resulting in a 5-year overall survival (OS) rate of 60–70%. However, it is estimated that up to 80% of patients with colorectal cancer liver metastases (CRLM) are not candidates for surgical resection at diagnosis. Furthermore, 50–60% of those who undergo treatment experience a recurrence [2]. When surgical options are not viable, local treatments include thermal methods (such as radiofrequency ablation [RFA] and laser-induced thermotherapy), transarterial chemoembolization (TACE), Y-90 radioembolization (RE), and various radiotherapy techniques, with stereotactic radiotherapy (SBRT) and high-dose rate (HDR) brachytherapy (BRT) being the most prominent [3]. The BRT also called interventional radiotherapy (I-RT) for liver metastases is a less commonly used option among local treatments. It is frequently compared to SBRT and proton therapy in terms of conformality, highlighting its potential to provide effective protection for critical organs in selected cases [4, 5]. One indication for brachytherapy in liver metastases is oligometastatic disease. Oligometastatic disease is characterized by a limited number of metastases and typically follows a less aggressive course. It represents an intermediate stage between locally advanced disease, where the primary intervention is local treatment supported by systemic therapy, and polymetastatic disease, where palliative systemic treatment is the main focus, with local treatment serving to alleviate symptoms or manage disease progression [6]. The aim of local ablative therapy in oligometastatic disease is to prolong progression-free survival (PFS), extend the time before the initiation of subsequent treatments, and ultimately improve overall survival (OS). However, there is a lack of randomized, prospective phase III clinical trials demonstrating the benefits of local treatments like brachytherapy for this patient group. Additionally, no reliable biomarkers exist to identify those patients who may benefit from such local treatment. Currently, the diagnosis of oligometastatic disease relies solely on imaging findings. A limited number of metastases seen through imaging may correspond to various clinical scenarios with differing prognoses, necessitating different treatment approaches.

In a study conducted by Guckenberger et al. [7], a meta-analysis identified 17 factors related to oligometastatic disease through a Delphi consensus. Consequently, nine distinct types of oligometastatic disease were identified, reflecting the complexity and variability within this concept. Among these, induced oligopersistence emerged as one of the more prevalent forms. This condition occurs when polymetastatic disease regresses following active systemic treatment, leaving only a few foci that meet the criteria for oligometastatic disease.

The present paper discusses the results of CT-BRT in cases of induced oligopersistence of liver metastases from colorectal cancer. The study also aimed to identify predictive factors for early response to this interventional radiotherapy approach.

## Materials and methods

This single-center cohort study retrospectively analyzed CRC patients with oligopersistence of liver metastases, who were treated with CT-BRT at first’s author brachytherapy department between 2017 and 2022. All patients had histopathologically confirmed CRC and underwent resection of the primary tumor. For those with rectal cancer, induction treatment included radiotherapy or radiochemotherapy, depending on clinical indications. All patients presented with metastases in various organs, excluding the central nervous system. Systemic treatment was administered in accordance with the prevailing National Comprehensive Cancer Network (NCCN) guidelines at the time of treatment. Key predictors for choosing systemic therapy included the KRAS gene mutation status, BRAF mutation status, and, in some patients, the assessment of DNA microsatellite instability (MSI). Based on this information, patients were qualified for subsequent lines of systemic treatment. In the first line, regimens utilizing fluoropyrimidine derivatives, combined with oxaliplatin or irinotecan, were administered alongside an EGFR inhibitor (for KRAS mutation wild type) or a VEGFR inhibitor (for KRAS mutated type). In the second line, a fluoropyrimidine-based regimen was used with oxaliplatin (if irinotecan was used in the first line) or with irinotecan (if oxaliplatin was used in the first line), in conjunction with an anti-VEGFR drug. Trifluridine/typiracil and regorafenib were used in the third line, while subsequent lines typically involved re-challenging with previously utilized drug combinations. Patients were eligible for pembrolizumab immunotherapy if they were confirmed to have microsatellite instability-high (MSI-H) or mismatch repair deficiency (dMMR).

Patients were eligible if they had received at least two lines of systemic treatment. Those with only one line were included if they declined further treatment or experienced unacceptable toxicity. All patients demonstrated partial regression of polymetastatic disease (intra- and extra-hepatic), with up to four persistent liver metastases that were stable for at least six months and amenable to brachytherapy. Regresję zmian w klatce piersiowej potwierdzano w tomografii komputerowej. Regression of thoracic lesions was confirmed by computer tomography (CT). Only patients who achieved complete regression of metastases in CT imaging were eligible for treatment. In cases where complete regression was not observed, positron emission tomography-computed tomography (PET-CT) was performed to confirm the absence of metabolic activity. For abdominal or pelvic lesions, eligibility for brachytherapy required either complete regression in CT, magnetic resonance imaging (MRI), or PET-CT imaging, or the absence of metabolic activity in MRI or PET-CT. No active extra-hepatic lesions were present at the time of study inclusion and therefore were not local treated with any methods (surgery, radiotherapy). Systemic treatment was discontinued prior to brachytherapy.

In addition to the primary eligibility conditions, patients had to meet specific inclusion criteria. These included a World Health Organization (WHO) performance status of less than 3 and a maximum tumor diameter of less than 10 cm. The total number of liver metastases could not exceed four, and the treatment had to be technically feasible, meaning there was no immediate proximity to large blood vessels. Laboratory parameters also had to fall within acceptable ranges, including a creatinine level below 2 mg/dl, hemoglobin (HGB) levels above 8 mg/dl, a white blood cell (WBC) count exceeding 2000/mm³, a neutrophil count (NEU) greater than 1500/mm³, and a platelet count (PLT) above 50,000/mm³. The international normalized ratio (INR) had to be less than 1.5. Additionally, levels of alanine aminotransferase (ALT), aspartate aminotransferase (AST), and total bilirubin (BIL) could not exceed 2.5 times the upper limit of normal.

Patients were excluded if the tumor location precluded the placement of the brachytherapy applicator. Other exclusion criteria included the presence of inflammatory conditions within the abdominal cavity and the proximity of critical structures that would prevent the delivery of the planned radiation dose.

### CT-BRT technique

The application was performed under either local or general anesthesia, with access through the intercostal space under CT guidance. Continuous fluoroscopic imaging was available (Siemens, Germany) using flexible needles approximately 1.7 mm in thickness and either 200–320 mm in length (Varian Medical Systems, Inc., USA). The gross tumor volume (GTV) consisted of the metastatic tumor visible on the CT scan during the application, along with the area identified in previous imaging studies. The clinical target volume (CTV) and planning target volume (PTV) were equal to the GTV. Planning was conducted using a three-dimensional treatment planning system, BrachyVision version 10 (Varian Medical Systems, Inc., USA). The treatment utilized a 24-channel GammaMedplus iX remote source loading device (Varian Medical Systems, Palo Alto, USA) equipped with an Ir192 source that had a nominal activity of 10 Ci and a diameter of 0.6 mm.

Three fraction doses were used: 15 Gy, 20 Gy, and 25 Gy. The selection of the dose depended on the location of critical structures surrounding the tumor and their tolerance to radiation. The dose was specified in an isodose of at least 95%. The main critical organ was the liver (D tolerance = D2/3 < 5 Gy), followed by the stomach (D tolerance = D1cc < 15 Gy), gallbladder (D tolerance = Dmax < 20 Gy), intestines (D tolerance = D1cc < 12 Gy), and kidney (V7 Gy < 2/3 volume).

### Follow-up and statistical analysis

The subsequent assessment, conducted after treatment, involved CT scans and, in some cases, MRI. To account for the phenomenon of pseudoprogression, the first evaluation occurred six months after brachytherapy. The dimensions and volumes of the lesions were consistently measured using the same imaging method as before the treatment. If progression was observed before four months and confirmed by a subsequent CT scan at least six months later, the date of progression was considered the date of detection of the first metastatic growth.

The maximum dimension of each metastasis was measured before and after brachytherapy, while the average dimension was calculated by dividing the total sum of these measurements by the number of metastases. The volume of all metastatic lesions was assessed both before and after brachytherapy, and the tumor burden score (TBS) was calculated using the formula: TBS2 = (maximum tumor diameter in cm)² + (number of tumors)².

The response was thoroughly assessed using the RECIST (Response Evaluation Criteria in Solid Tumors) 1.1 scale, based on the evaluation of target lesions as defined by the RECIST criteria. The first response assessment with CT of the chest, abdomen and pelvis was performed 3–4 months after CT-BRT and was performed according to RECIST 1.1 criteria. The second assessment was performed at 6–7 months and was chosen to assess response within the liver. Before this time, there was a possibility of misinterpretation of the diagnostic tests, mainly in favour of the diagnosis of progression (PD), due to the frequent presence of central necrosis, hypointense rim, inflammation and/or oedema in and around the metastasis (21, 22). If PD was present at the first and second diagnostic assessments, it was counted from the first diagnostic scan. Follow-up was performed at regular intervals of 3–4 months with the same diagnostic tests. The study endpoints included OS, defined as survival time from brachytherapy to death, and PFS, defined as survival free from tumor progression at any site, including areas outside the treated region. The time free from progression of a treated lesion was not defined separately, as such progression was associated with a change in treatment strategy, which could affect the recorded duration of local progression-free status.

Survival analysis among different study groups was compared using the log-rank test, and Kaplan-Meier curves were generated. Cox proportional hazards regression analysis was employed to analyze prognostic factors for local PFS and OS [8]. Non-normality of the data was identified using the Shapiro-Wilk test, and data comparisons were made using non-parametric tests (Mann-Whitney U test) [8]. The values of the parameters examined to predict the occurrence of an endpoint were evaluated using receiver operating characteristic (ROC) curve analysis. A p-value of less than 0.05 was considered statistically significant. The statistical analysis was performed using the R programming language (version 4.2.0, Lucent Technologies, USA).

Toxicity assessment was performed using the Common Terminology Criteria for Adverse Events (CTCAE) scale. The study received approval from the local bioethical committee (No. LIL-KB-20/2014), and written consent was obtained from each patient. The manuscript was prepared in accordance with the STROBE (Strengthening the Reporting of Observational Studies in Epidemiology) guidelines (Supplementary Material 2) [9].

## Results

A total of 68 out of 270 colorectal cancer (CRC) patients with liver metastases treated by CT-CRT met the inclusion criteria and were included in the analysis. Basic characteristics of included patients and selected variables are shown in Table 1. The median follow-up time was 17 months (3–30). The median OS was 16 months (6–30). The OS rates at 6, 12, and 24 months were 99%, 77%, and 18%, respectively (Fig. 2). The median PFS was 13 months (2–27), with PFS rates at 6, 12, and 24 months being 85%, 51%, and 5%, respectively (Fig. 3).

**Table 1:** Clinical characteristics of the selected patients, treatment and treatment response

Variable	Number of patients (percentage)	Median (range)
Gender		
- men	36 (53%)	-
- women	32 (47%)	
Age		
≤ 50 years	26 (37%)	
51–70 years	40 (59%)	
≥ 70 years	3 (4%)	
Tumour location		
- rectum	25 (63%)	
- colon		
· Sigmoid	12 (18%)	
· Descending colon	6 (9%)	
· Transverse colon	9 (13%)	
· Ascending colon	15 (22%)	
Number of liver metastases treated		2 (1–4)
1	29 (43%)	
2	24 (35%)	
3	6 (9%)	
4	8 (12%)	
Number of applicators used		2 (1–8)
1	20 (29%)	
2	19 (28%)	
3	10 (15%)	
4	8 (12%)	
5	8 (12%)	
6	1 (1%)	
7	0	
8	1 (1%)	
RAS		
(+)	17 (25%)	
(-)	49 (72%)	
Lung metastases		
Yes	21 (31%)	
No	47 (69%)	
Metastases to peritoneum and/or abdominal or pelvic lymph nodes		
Yes	2 (3%)	
No	66 (97%)	
Baseline T		
1	3	
2	6	
3	43	
4	15	
Baseline N		
0	11	
1	22	
2	32	
3	3	
Degree of malignancy		
G1	23	
G2	35	
G3	9	
Number of systemic therapy lines at brachytherapy		3 (1–5)
1	1	
2	27	
3	25	
4	13	
5	1	
Maximal diameter of the largest tumor before BRT (cm)		4 (1–10)
Maximal diameter of the largest tumor after BRT (cm)		3 (0–9)
TBS before BRT		4,47 (1.41–10.77)
TBS after BRT		3,6 (1-9.84)
TBS difference		0,86 (0.82-4,04)
Medial diameter of the one tumor before BRT (cm)		3 (1–7)
Medial diameter of the one tumor before BRT (cm)		2 (0–6)
Difference of medial dimensions before and after BRT		1 (-1–4,9)
Volume of the medial liver tumour before brachytherapy (cm3)		27 (1-343)
Volume of the medial liver tumour after brachytherapy (cm3)		8 (0-216)
Volume of all liver metastases before brachytherapy (cm3)		54 (1-1372)
Volume of all liver metastases after brachytherapy (cm3)		16 (0-864)
Difference of volume all metastases before and after BRT (cm3)		21 (181–872)
Prescribed dose (Gy)		20 (15–25)

**Fig. 1:**
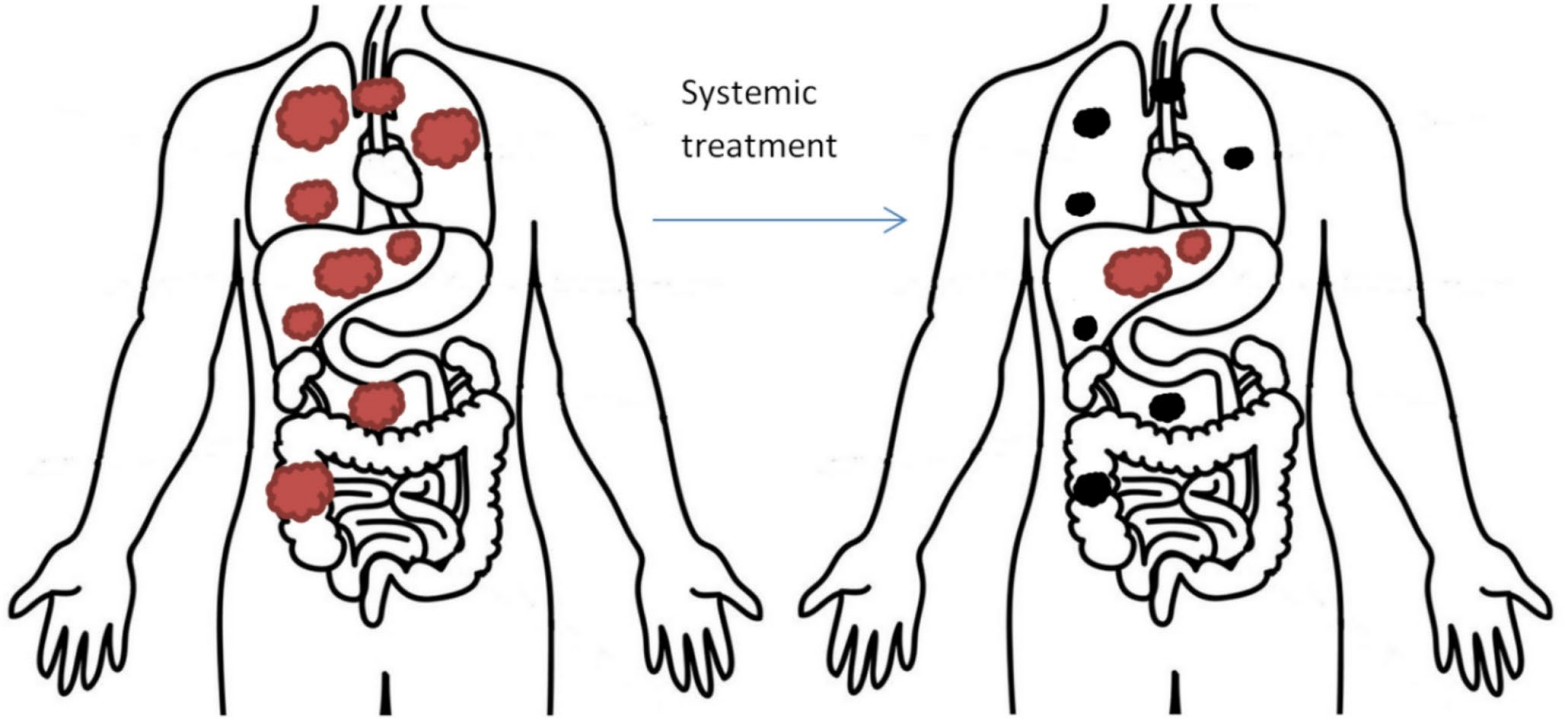
Illustration of the concept of induced oligopersistence which refers to the emergence of a few metastases in a patient who is otherwise responding well to treatment. The left image demonstrates polymetastatic disease with multiple active tumor foci in the liver and beyond (indicated in red). The right image shows polymetastatic disease after systemic treatment, with persistent metastatic foci that have regressed (indicated in black). Oligopersistence is depicted (indicated in red) in areas where metastatic lesions were previously present

**Fig. 2:**
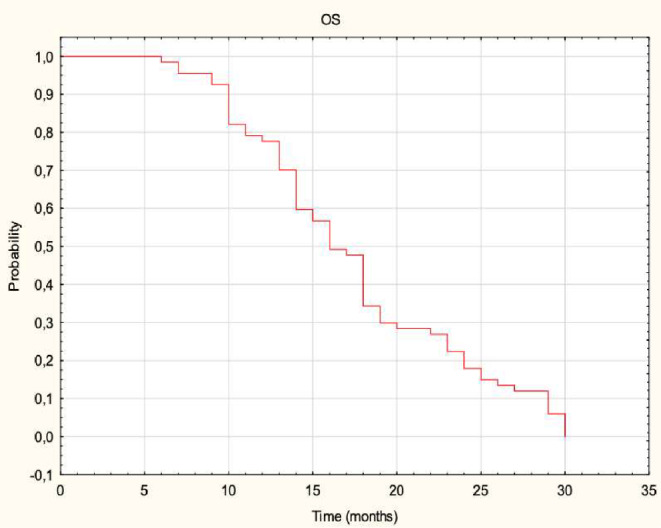
The Overall survival in the entire cohort. Kaplan-Meier curve illustrating overall survival (OS) for patients treated with CT-guided high-dose-rate brachytherapy (CT-BRT) for colorectal cancer liver metastases. The x-axis represents time in months, while the y-axis indicates the probability of survival. The stepwise decline reflects censoring and survival events over time. Median OS and survival probabilities at 6, 12, and 24 months are provided in the results section

**Fig. 3:**
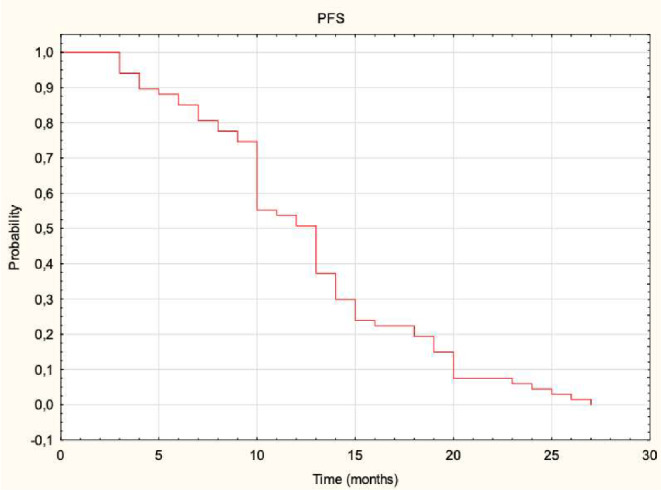
The progression-free survival in the entire cohort. Kaplan-Meier curve illustrating progression-free survival (PFS) for patients treated with CT-guided high-dose-rate brachytherapy (CT-BRT) for colorectal cancer liver metastases. The x-axis represents time in months, while the y-axis indicates the probability of remaining progression-free. The stepwise decline reflects disease progression or censoring over time. Median PFS and survival probabilities at 6, 12, and 24 months are detailed in the results section

In the univariate Cox analysis, several factors significantly affected OS, including: the presence of abdominal, pelvic, and peritoneal lymph node metastasis; the size of the largest metastasis after brachytherapy; the variations in TBS; the mean size of a single metastatic lesion; the type of RECIST response; ORR; and the mean volume of metastases after brachytherapy. Factors affecting PFS included: the size of the largest metastasis after brachytherapy; TBS difference (borderline significance); mean size of a single metastatic lesion; type of RECIST response; percentage of ORR; and the volume of metastases after brachytherapy. (Supplementary material Table S1, Table S2).

In the multivariate analysis, factors influencing OS were the presence of abdominal, pelvic, or peritoneal lymph node metastasis and ORR. For PFS, significant factors included TBS difference and the mean size of a single metastatic lesion after brachytherapy.

In our analyzed patient cohort, complete response (CR) was observed in 10 patients (7%), partial response (PR) in 24 patients (35%), stable disease (SD) in 30 patients (44%), and progressive disease (PD) in 4 patients (6%). The disease control rate (DCR) and ORR were 94% and 41%, respectively.

**Fig. 4:**
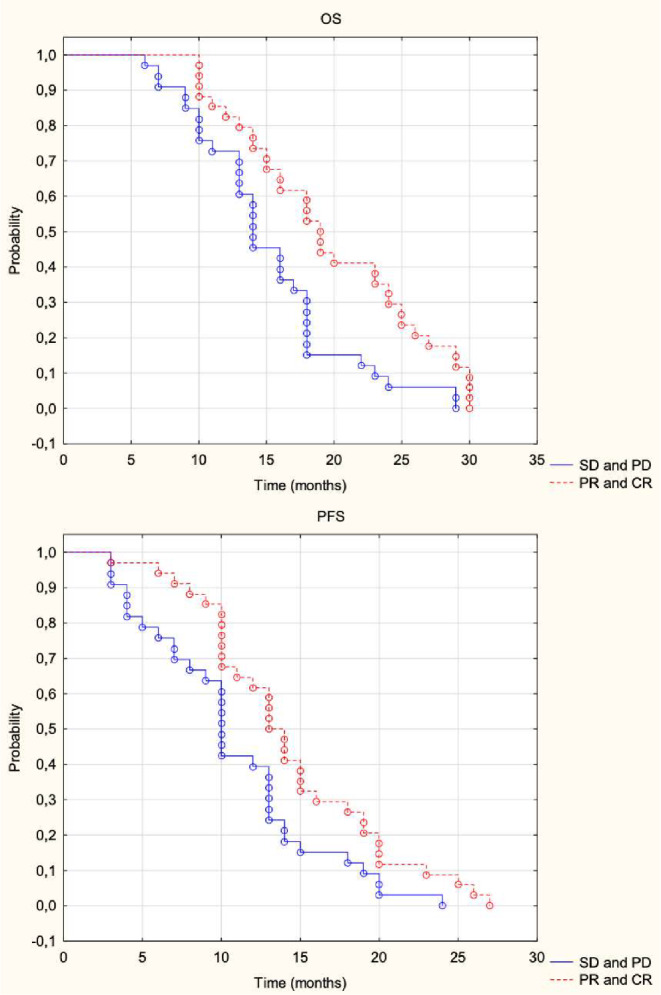
Kaplan-Meier curves for overall survival (OS, top) and progression-free survival (PFS, bottom) based on RECIST 1.1 (Response Evaluation Criteria in Solid Tumors) response. Red dashed lines indicate partial response (PR) and complete response (CR), and blue solid lines represent stable disease (SD) and progressive disease (PD)

The OS in patients with ORR was 19 months; in the SD or PD group, it was 14 months. The OS rates for patients with ORR at 6, 12, and 24 months were 100%, 82%, and 29%, respectively. For the SD or PD group, these rates were 97%, 73%, and 6% (*p* = 0.004). The median PFS in patients with ORR was 13 months, while in the SD or PD group, it was 10 months. The PFS rates for patients with ORR at 6, 12, and 24 months were 94%, 62%, and 9%, respectively. In the SD or PD group, they were 76%, 39%, and 0% (*p* = 0.019). Figure 4.

**Fig. 5:**
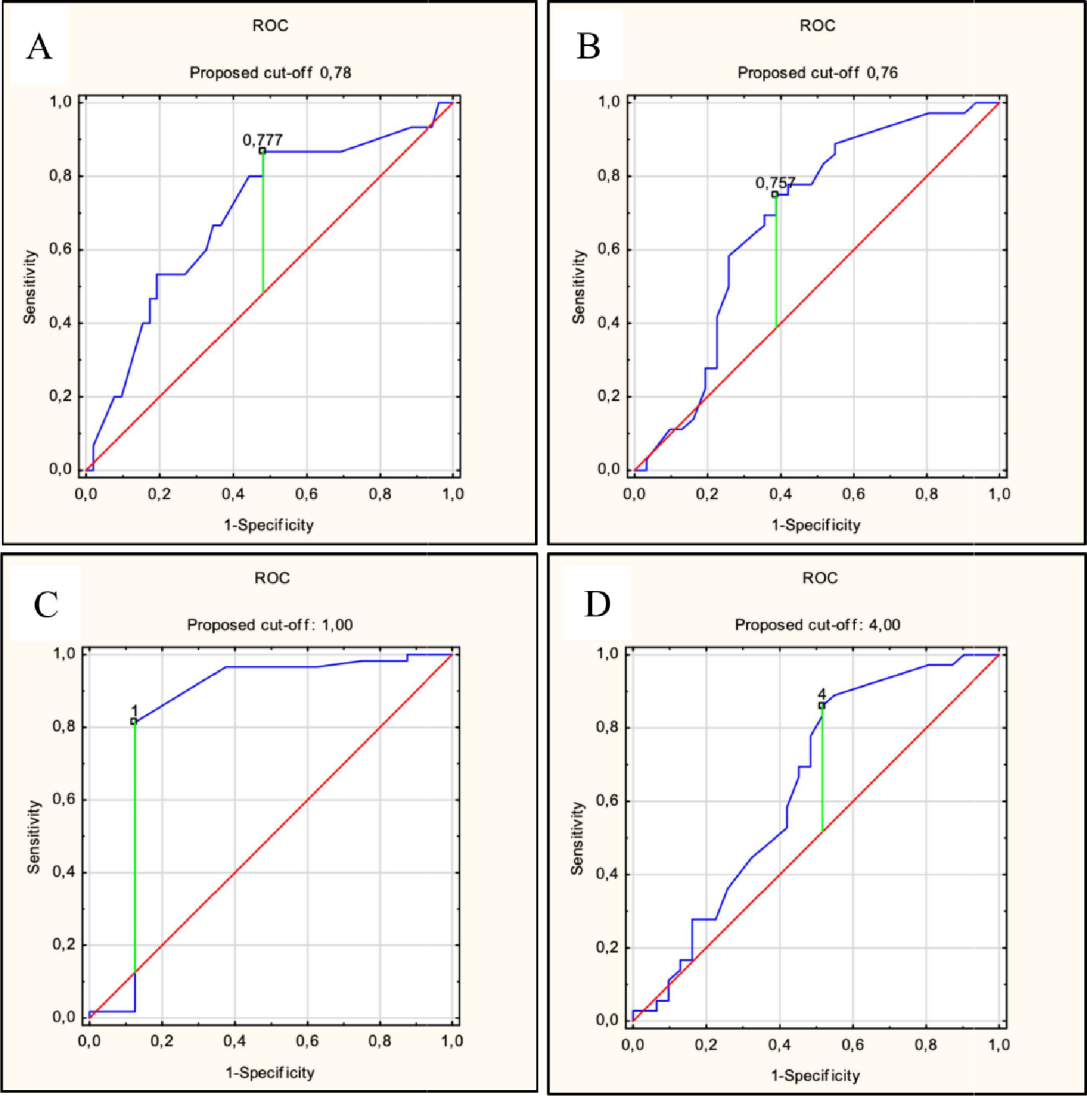
Receiver Operating Characteristic (ROC) Curves. The ROC curves show optimal cutoff values for clinical endpoints and parameters: (**A**) Tumor burden score (TBS) value change predicting overall survival (OS) at 24 months, cutoff 0.78., (**B**) TBS value change predicting progression-free survival (PFS) at 12 months, cutoff 0.76., (**C**) Volume change predicting PFS at 6 months, cutoff 1.00., (**D**) Volume change predicting PFS at 12 months, cutoff 4.00. The blue line represents the ROC curve, the red diagonal line indicates a random classifier (AUC = 0.5), and the green vertical line marks the optimal sensitivity and specificity

**Fig. 6:**
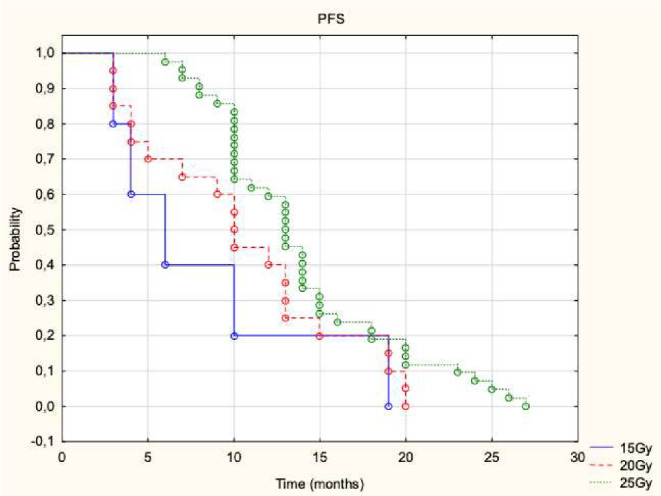
Kaplan-Meier Progression-Free Survival (PFS) Curves. The Kaplan-Meier curves display progression-free survival probabilities over time (in months) for patients receiving different radiation doses: Blue solid line: 15 Gy, Red dashed line: 20 Gy, Green dotted line: 25 Gy. The curves illustrate the impact of dose escalation on PFS, with probabilities decreasing over time across all dose groups

Cutoff values for TBS difference and total metastasis volume difference were established for OS and PFS parameters. For TBS difference, significant cutoff values were observed for 24-month OS (0.777; sensitivity 85%, specificity 51%, AUC = 0.699, *p* = 0.011) and PFS at 6 months (0.09; sensitivity 81%, specificity 87%, AUC = 0.842, *p* = 0.003) and 12 months (0.76; sensitivity 75%, specificity 62%, AUC = 0.683, *p* = 0.008). For total metastasis volume difference, significant cutoff values were identified for PFS at 6 months (1 ml; sensitivity 81%, specificity 88%, AUC = 0.836, *p* = 0.003) and 12 months (4 ml; sensitivity 84%, specificity 49%, AUC = 0.642, *p* = 0.043). These results are illustrated in Fig. 5 with additional parameters shown in Supplementary Materials Table S3. The dosage did not significantly impact OS but influenced PFS. Median OS for 15 Gy, 20 Gy, and 25 Gy was 13, 16, and 17 months, respectively (*p* = 0.359). Median PFS for these doses was 6, 10, and 13 months, respectively (*p* = 0.027). PFS rates at 6, 12, and 24 months for 15 Gy, 20 Gy, and 25 Gy were 40%, 20%, and 0%; 70%, 40%, and 0%; and 98%, 60%, and 7%, respectively, as shown in Fig. 6.

**Fig. 7:**
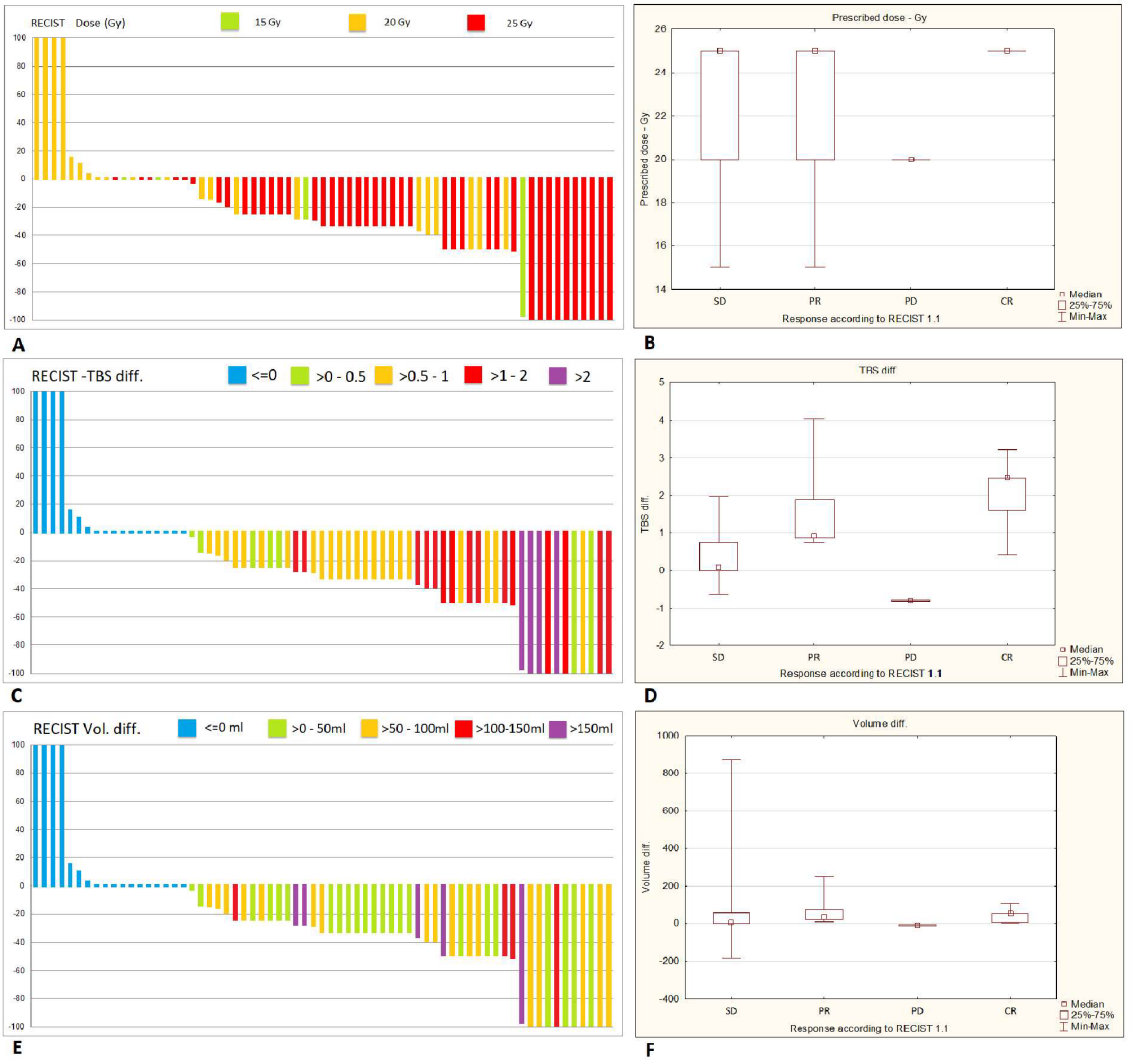
The Relationship Between Response Rate and Administered Dose, TBS Difference, and Volume Change. (**A**) Waterfall plot showing RECIST 1.1 response rates categorized by prescribed doses (15 Gy, 20 Gy, 25 Gy)., (**B**) Box plot illustrating the distribution of prescribed doses across RECIST 1.1 response categories: stable disease (SD), partial response (PR), progressive disease (PD), and complete response (CR)., (**C**) Waterfall plot showing RECIST 1.1 response rates in relation to changes in tumor burden score (TBS), categorized by TBS difference ranges (≤ 0, > 0–0.5, > 0.5–1, > 1–2, > 2)., (**D**) Box plot of TBS differences across RECIST 1.1 response categories., (**E**) Waterfall plot depicting RECIST 1.1 response rates based on total volume change of liver metastases, categorized by volume differences (≤ 0 mL, > 0–50 mL, > 50–100 mL, > 100–150 mL, > 150 mL)., (**F**) Box plot showing volume differences across RECIST 1.1 response categories

The type of treatment response was associated with the delivered dose (Z = 8.415, *p* = 0.015). The median dose for the CR, PR, and SD groups was 25 Gy, while for the PD group, it was 20 Gy. Similarly, changes in TBS differed according to the type of treatment response (Z = 15.088, *p* = 0.017), with the median TBS differences being 0.87, 0.06, 0.88, and 2.46 for CR, PR, SD, and PD, respectively. The volume change of all liver metastases also varied by treatment response (Z = 11.347, *p* = 0.01), with median volume differences of 14 cm³ for CR, 7 cm³ for PR, 39 cm³ for SD, and 54 cm³ for PD. (Fig. 7; Table 1).

The brachytherapy was well tolerated. The predominant acute complications included a transient increase in aminotransferase levels observed in 47 patients (69%) within the first two weeks following treatment, injection site pain during the first three days post-treatment in 32 patients (47%), and bleeding at the injection site in seven patients (10%). All complications were graded G1-G2, with no cases of G3 or higher acute toxicity. Among the late toxicities, two patients experienced chronic intercostal nerve neuropathy at the injection site, classified as grade G2. There were no late toxicities of grade G3 or higher.

## Discussion

Oligopersistent disease refers to a specific type of response to systemic treatment. The presence of surviving cell clones following drug treatment is a significant factor contributing to treatment failure. Several reasons account for this condition, including slow proliferation, flexible energy consumption, adaptation to the microenvironment, and phenotypic plasticity [10]. The presence of oligopersistent disease at the macroscopic level is measurable and can be assessed with imaging studies. The primary tool for evaluating treatment response is the RECIST scale [11]. Although numerous modifications have been made to the RECIST scale in relation to new forms of treatment such as immunotherapy, the scale remains widely used [12]. Despite certain criticisms, many studies have validated the utility of this scale in predicting subsequent prognosis, including in cases of CRC [13, 14]. The main criterion for evaluating response in the RECIST scale is the size of the lesion. Aside from achieving a CR or demonstrating a lack of metabolic activity in imaging studies, all other types of response are considered persistent disease. Our analysis showed that greater tumor reduction or CR on the RECIST scale significantly improves OS and PFS compared to stable disease or progression. Significant differences were noted in both univariate and multivariate analyses for OS and in univariate analyses for PFS, highlighting the benefit associated with reduced tumor dimensions or complete regression. However, additional factors can influence the relationship between tumor reduction and patient prognosis. One such factor is the volume of all metastatic lesions. Research indicates that tumor volume, reflecting overall tumor mass, significantly impacts survival and recurrence rates [15]. This study identified a cutoff value of 100 ml of metastatic volume for five-year survival (41% vs. 67%). Moreover, a pre-treatment to post-treatment volume difference of at least 10% had a statistically significant effect on relapse-free survival (RFS) [16]. It was also shown that for a total tumor volume (TTV) of 3 ml, 5-year OS was 72%, whereas it was only 7% for a volume of 300 ml. The findings suggested that both baseline tumor volume and the difference in tumor volume after treatment serve as predictors for early recurrence in both univariate and multivariate analyses [17]. There are no data on the use of brachytherapy for the treatment of metastases in this indication. Previous work has analysed patients with multiple indications together, in different clinical cases, without including patients with oligopersistence disease [3].

Our cohort’s univariate analysis indicated that the mean size and volume of metastasis after treatment influenced OS and PFS. Furthermore, we found that the type of RECIST response correlates with the post-treatment volume difference, suggesting potential benefits in OS and PFS from reduced volume of liver metastases after local treatment.

Another crucial parameter that correlates with prognosis in patients with liver metastases is tumor burden score (TBS), first described in 2018 by Sasaki et al. [18]. The TBS was found to be a good indicator of the relationship between tumor morphology and long-term survival outcomes in patients after resection of CRC metastases. According to a study by Peng et al., TBS also predicts conversion to surgery for patients with unresectable liver metastases after systemic treatment [19]. Patients exhibiting a high TBS prior to treatment had a worse ORR to treatment and a lower conversion rate. However, there are currently no studies employing this indicator to forecast prognosis after radiation therapy, particularly brachytherapy.

Our results demonstrated a dependence of the TBS difference on the RECIST response, along with a significant effect of TBS on OS in univariate analysis, and on PFS in both univariate and multivariate analyses.

The cutoff values for tumor volume reduction and TBS have clinical implications. Although, due to the sample size and retrospective nature of the study, not all values at subsequent time points for OS and PFS reached clinical significance, a discernible trend was observed, warranting further investigation. A reduction in tumor volume by ~ 6 mL and 38 mL was associated with an increased probability of 12-month and 24-month OS, respectively (not statistically significant but approaching significance). Similarly, a tumor volume reduction of 1 mL and 4 mL significantly increased the likelihood of 6-month and 12-month PFS, respectively.

Additionally, a TBS reduction of 0.76 and 0.78 was associated with 12-month and 24-month OS, respectively (statistically significant only for 24-month OS). Likewise, a TBS reduction of 0.09, 0.76, and 2.47 was associated with 6-month, 12-month, and 24-month PFS, respectively (statistically significant only for 12-month PFS).

The median OS in the analyzed group of patients was 16 months, and the median PFS was 13 months. These results pertain to advanced patients with baseline polymetastatic disease, in whom oligopersistent disease in the liver involved up to four metastases, up to 10 cm, after an average of three lines of systemic therapy. Although the findings indicate a poor prognosis in this group, the efficacy of brachytherapy aligns with results found in other studies [3].

There is a lack of randomized trials in this group of patients; the only randomized phase II study, SABR-COMET, is on stereotactic body radiotherapy (SBRT) and shows a benefit from the addition of SBRT to the standard of care (SOC) in oligometastatic cancer patients. However, primaries were included in this study, with excellent OS results in both the control and study groups. The CRC accounted for 14% of patients in the SABR group [20]. In our cohort, a significant factor affecting PFS was metastasis to abdominal, pelvic, or peritoneal lymph nodes. This is due to the increased tumor mass and the limited ability to assess the complete regression of peritoneal metastases on CT [21]. Once again, the effect of high doses on local treatment efficacy was also confirmed, indicating the need to try further dose escalation while maintaining high application precision and protecting critical organs [4, 5, 22, 23].

To the best of our knowledge, there are no articles specifically addressing oligopersistent liver metastases, particularly no articles comparing the efficacy of local treatments in such settings. Even comparing the local control rates of different methods for unselected liver metastases is challenging because each method treats varying volumes and tumor characteristics. Stereotactic Body Radiation Therapy (SBRT) generally has a 1-, 2-, and 3-year actuarial local control rate of 93%, 89%, and 86%, respectively, according to a meta-analysis [24]. In another recent meta-analysis of 3,101 patients confirmed 1- and 3-year LC rates of 85% and 68%, supporting SBRT’s inclusion in ASCO guidelines [25, 26]. However, liver motion requires CTV/PTV margins, increasing normal tissue irradiation, whereas CT-BRT eliminates this need, preserving healthy liver [25]. For large metastases (> 3 cm), SBRT outperforms RFA, MWA, and embolization, but dose constraints remain a key predictor of LC [25]. CT-BRT delivers > 1000 Gy near catheters with a steep dose fall-off, avoiding SBRT’s limitations. In our study, dose was limited only by OAR proximity, not lesion size, underscoring a key advantage of CT-BRT. The local control rate by Radiofrequency Ablation (RFA) and Microwave Ablation (MWA) varied widely among the studies, ranging approximately 50–90% [27].

Our work stands as the first, comprehensive analysis, underscoring the crucial role of reducing the size of liver metastases, achieved through CT-BRT, in patients with induced oligopersistance CRC.

This pioneering study provides valuable insights into the potential of CT-guided HDR brachytherapy (CT-BRT) for oligopersistent liver metastases in CRC patients. While our findings suggest improved PFS and OS, the impact of initial tumor burden and treatment of oligoprogression remains an area requiring prospective evidence to confirm its oncological benefits. Future studies should prioritize randomized trials and investigate the integration of CT-BRT into multimodal strategies for broader applicability and validation.

A limitation of this study is its retrospective nature, conducted at a single center on a relatively small cohort of 68 patients, without a control group. This is primarily due to the limited adoption of this treatment method, which requires specialized training, experience, and advanced technical capabilities. However, the study was conducted on a homogeneous group of patients with consistent indications for local treatment in the setting of oligopersistence. The authors acknowledge that ethnic, genetic, and geographic factors may influence the study outcomes. Additionally, quality-of-life analysis was not performed; however, toxicity assessment indicates that the treatment was well tolerated. Another limitation is the reliance on RECIST criteria for response evaluation in follow-up assessments. Nevertheless, RECIST remains the standard criterion for qualifying patients for systemic therapy, which was the primary treatment in cases of disease progression. Despite these limitations, this study represents the largest analysis conducted on a homogeneous patient cohort, presenting a novel therapeutic approach. As such, it may serve as a foundation for future randomized trials.

## Conclusions

CT-BRT in induced oligopersistence of CRC liver metastases has shown promising results, with high disease control rates and very low toxicity. The RECIST response and size reduction have been linked to prolonged OS and PFS, offering hope for improved patient outcomes. The tumor burden score has emerged as a reliable predictor for PFS and OS, further underlining the potential impact of our findings.

## Electronic supplementary material

Below is the link to the electronic supplementary material.

Supplementary Material 1

Supplementary Material 2

## Data Availability

Detailed patient data and the results of statistical analyses used in this study will be made available upon reasonable request to the corresponding author, in accordance with data protection regulations and applicable legal requirements.

## Abbreviations

ALT: Alanine Aminotransferase
AST: Aspartate Aminotransferase
BIL: Bilirubin
BRT: Brachytherapy
CRC: Colorectal Cancer
CRLM: Colorectal Liver Metastases
CR: Complete Response
CTV: Clinical Target Volume
CT-BRT: Computed Tomography-Guided Brachytherapy
CTCAE: Common Terminology Criteria for Adverse Events
DCR: Disease Control Rate
dMMR: Mismatch Repair Deficiency
EGFR: Epidermal Growth Factor Receptor
GTV: Gross Tumor Volume
HGB: Hemoglobin
HDR: High-Dose Rate
I-RT: Interventional Radiotherapy
INR: International Normalized Ratio
KRAS: Kirsten Rat Sarcoma Viral Oncogene Homolog
MSI: Microsatellite Instability
MSI-H: Microsatellite Instability-High
MRI: Magnetic Resonance Imaging
MWA: Microwave Ablation
NCCN: National Comprehensive Cancer Network
NEU: Neutrophil Count
ORR: Overall Response Rate
OS: Overall Survival
PD: Progressive Disease
PET-CT: Positron Emission Tomography-Computed Tomography
PFS: Progression-Free Survival
PLT: Platelet Count
PR: Partial Response
PTV: Planning Target Volume
RFA: Radiofrequency Ablation
RE: Radioembolization
RECIST: Response Evaluation Criteria in Solid Tumors
ROC: Receiver Operating Characteristic
RFS: Relapse-Free Survival
SBRT: Stereotactic Body Radiotherapy
SD: Stable Disease
SOC: Standard of Care
STROBE: Strengthening the Reporting of Observational Studies in Epidemiology
TACE: Transarterial Chemoembolization
TBS: Tumor Burden Score
TTV: Total Tumor Volume
VEGFR: Vascular Endothelial Growth Factor Receptor
WBC: White Blood Cell Count
WHO: World Health Organization
Y-90: Yttrium-90 Radioembolization

## Declarations

### Accountability

Each author agrees to be accountable for all aspects of the work, ensuring that questions related to accuracy or integrity are appropriately investigated and resolved.
