# Supplementary material for: EORTC/ESTRO defined induced oligopersistence of liver metastases from colorectal cancer - outcomes and toxicity profile of computer tomography guided high-dose-rate brachytherapy
Source: Clin Exp Metastasis. 2025 May 12;42(3):29. doi: 10.1007/s10585-025-10348-z (PMC12069121; doi:10.1007/s10585-025-10348-z)
Supplement: Supplementary file 1 — Supplementary Material 1 [file 10585_2025_10348_MOESM1_ESM.docx]

**Supplementary material 1.**

**Table S1.**

Results of Univariate and Multivariate Analyses for Overall Survival

The table presents the results of univariate and multivariate analyses assessing factors associated with overall survival (OS) in 68 patients treated with brachytherapy for colorectal cancer (CRC) liver metastases. The analyses include hazard ratios (HR) with corresponding 95% confidence intervals (CI) and p-values for each evaluated parameter. The univariate analysis examines individual factors such as the presence of lung metastases, maximal tumor dimensions before and after brachytherapy (BRT), Tumor Burden Score (TBS), response to treatment according to RECIST 1.1 (Response Evaluation Criteria in Solid Tumors), and the overall response rate (ORR). The multivariate analysis identifies independent predictors of OS, with key variables adjusted for confounding effects. The results highlight the clinical significance of certain tumor characteristics and treatment response metrics in predicting survival outcomes.

| N = 68 | p | HR | HR 95% lower | HR 95% upper |
| --- | --- | --- | --- | --- |
| **Univariate analysis** | | | | |
| Lung metastases | 0,845851 | 1,053151 | 0,624824 | 1,775102 |
| Metastases to lymph nodes in the abdominal cavity and/or pelvis (no-0, yes-1) | 0,002999 | 0,111111 | 0,021115 | 0,45407 |
| Maximal diameter of the largest tumour before BRT | 0,978637 | 1,002046 | 0,862784 | 1,163787 |
| Maximal diameter of the largest tumour after BRT | 0,028098 | 1,144480 | 1,014612 | 1,290971 |
| TBS before BRT | 0,943188 | 0,994983 | 0,866436 | 1,142601 |
| TBS after BRT | 0,130274 | 1,107268 | 0,970352 | 1,263502 |
| TBS difference | 0,007318 | 0,709075 | 0,551554 | 0,911583 |
| Medial diameter of the one tumour before BRT | 0,814017 | 1,022901 | 0,847042 | 1,235272 |
| Medial diameter of the one tumour before BRT | 0,034513 | 1,203236 | 1,013581 | 1,428376 |
| Difference of medial dimensions before and after | 0,018165 | 0,764855 | 0,612335 | 0,955365 |
| Response according to RECIST 1.1 | 0,018767 | 0,725568 | 0,555255 | 0,948121 |
| ORR | 0,00588 | 0,488033 | 0,292935 | 0,813066 |
| Volume of the medial liver tumour before brachytherapy (cm3) | 0,586617 | 1,000968 | 0,997482 | 1,004467 |
| Volume of the medial liver tumour after brachytherapy (cm3) | 0,04486 | 1,005075 | 1,000116 | 1,010059 |
| Volume of all liver metastases before brachytherapy (cm3) | 0,509940 | 1,000352 | 0,999306 | 1,001399 |
| Volume of all liver metastases after brachytherapy (cm3) | 0,053017 | 1,00163 | 0,999979 | 1,003283 |
| Difference of volume all metastases before and after BRT | 0,483552 | 0,999087 | 0,996537 | 1,001644 |
| Prescribed dose | 0,423212 | 0,892312 | 0,675176 | 1,179279 |
| Number of lines of chemotherapy | 0,644496 | 1,062605 | 0,821022 | 1,375273 |
| **Multivariate analysis** | | | | |
| Metastases to lymph nodes in the abdominal cavity and/or pelvis | 0,001172 | 13,27199 | 2,785076 | 63,24629 |
| ORR | 0,003685 | 0,46948 | 0,281824 | 0,78209 |

**Table S2.**

Results of Univariate and Multivariate Analyses for Progression-Free Survival (PFS)

This table summarizes the univariate and multivariate analyses evaluating factors associated with progression-free survival (PFS) in 68 patients with colorectal cancer (CRC) liver metastases treated with brachytherapy (BRT). Hazard ratios (HR) with 95% confidence intervals (CI) and p-values are presented for each variable. The univariate analysis includes parameters such as lung metastases, tumor dimensions before and after BRT, Tumor Burden Score (TBS) before and after BRT, differences in TBS and tumor dimensions, response according to RECIST 1.1 (Response Evaluation Criteria in Solid Tumors), overall response rate (ORR), and the volume of liver metastases. The multivariate analysis identifies independent predictors of PFS, considering the effects of TBS difference and tumor dimensions after BRT. These findings underscore the relevance of specific tumor characteristics and treatment response in determining disease progression.

| N = 68 | p | HR | HR 95% lower | HR 95% upper |
| --- | --- | --- | --- | --- |
| **Univariate analysis** | | | | |
| Lung metastases | 0,977162 | 0,992366 | 0,587252 | 1,676947 |
| Metastases to lymph nodes in the abdominal cavity and/or pelvis (no-0, yes-1) | 0,135104 | 0,33249 | 0,07844 | 1,40935 |
| Maximal diameter of the largest tumour before BRT | 0,643031 | 1,032848 | 0,900903 | 1,184118 |
| Maximal diameter of the largest tumour after BRT | 0,004579 | 1,176963 | 1,051595 | 1,317277 |
| TBS before BRT | 0,774678 | 1,018738 | 0,89714 | 1,156819 |
| TBS after BRT | 0,053667 | 1,12931 | 0,998087 | 1,277786 |
| TBS difference | 0,002627 | 0,657761 | 0,500662 | 0,864156 |
| Medial diameter of the one tumour before BRT | 0,330985 | 1,094637 | 0,912214 | 1,313542 |
| Medial diameter of the one tumour before BRT | 0,001459 | 1,315634 | 1,111154 | 1,557744 |
| Difference of medial dimensions before and after | 0,002789 | 0,692226 | 0,543928 | 0,880957 |
| Response according to RECIST 1.1 | 0,025365 | 0,756286 | 0,59203 | 0,966114 |
| ORR | 0,028254 | 0,572929 | 0,34833 | 0,942349 |
| Volume of the medial liver tumour before brachytherapy (cm3) | 0,337104 | 1,001592 | 0,998344 | 1,004851 |
| Volume of the medial liver tumour after brachytherapy (cm3) | 0,00645 | 1,006939 | 1,001942 | 1,011961 |
| Volume of all liver metastases before brachytherapy (cm3) | 0,647286 | 1,000225 | 0,999261 | 1,001190 |
| Volume of all liver metastases after brachytherapy (cm3) | 0,030973 | 1,001714 | 1,000157 | 1,003274 |
| Difference of volume all metastases before and after BRT | 0,331453 | 0,998656 | 0,99595 | 1,00137 |
| Prescribed dose | 0,02656 | 0,915195 | 0,846263 | 0,989743 |
| Number of lines of chemotherapy | -0,042635 | 0,146669 | -0,330101 | 0,244832 |
| **Multivariate analysis** | | | | |
| TBS difference | 0,016922 | 1,248984 | 1,040689 | 1,498971 |
| Medial diameter of the one tumour after BRT | 0,026001 | 0,720229 | 0,539493 | 0,961513 |

**Table S3**

Table summarizes the cutoff values, sensitivity, specificity, AUC (Area Under the Curve), and p-values for TBS (tumor burden score) difference and volume difference parameters associated with overall survival (OS) and progression-free survival (PFS) at various time points (6, 12, and 24 months). Statistically significant results (p < 0.05) are highlighted, reflecting their potential predictive value

| Parameter | Cutoff Value | Sensitivity | Specificity | AUC | p-value |
| --- | --- | --- | --- | --- | --- |
| TBS difference, 12 months OS | 0.757 | 66% | 70% | 0.606 | 0.237 |
| TBS difference, 24 months OS | 0.777 | 85% | 51% | 0.699 | **0.011** |
| TBS difference, 6 months PFS | 0.09 | 81% | 87% | 0.842 | **0.003** |
| TBS difference, 12 months PFS | 0.76 | 75% | 62% | 0.683 | **0.008** |
| TBS difference, 24 months PFS | 2.472 | 50% | 92% | 0.683 | 0.219 |
| Volume difference, 12 months OS | 5.58 ml | 70% | 57% | 0.614 | 0.169 |
| Volume difference, 24 months OS | 38 ml | 66% | 57% | 0.614 | 0.145 |
| Volume difference, 6 months PFS | 1 ml | 81% | 88% | 0.836 | **0.003** |
| Volume difference, 12 months PFS | 4 ml | 84% | 49% | 0.642 | **0.043** |
| Volume difference, 24 months PFS | 4.63 ml | 99% | 33% | 0.575 | 0.533 |
